# Differences Between a Monoclonal Anti-NC1 Antibody (12D) That Selectively Stains Injured Kidneys and Other Anti-NC1 Antibodies

**DOI:** 10.7759/cureus.107782

**Published:** 2026-04-27

**Authors:** Tsukao Yokoyama, Yoshiaki Tabuchi, Shunji Hattori

**Affiliations:** 1 Department of Research, Collagen Research Center, Kiyose, JPN; 2 Graduate School of Innovative Life Science, University of Toyama, Toyama, JPN; 3 Institute for Protein Research, Matrixome, Osaka University, Suita, JPN

**Keywords:** 2-mercaptoethanol (2me), coomassie brilliant blue (cbb), disulfide bond, glomerulonephritis, indirect immunohistochemistry, monoclonal anti-nc1 antibody (12d), sds–page, type iv collagen nc1, western blot, α5 chain

## Abstract

Background

Type IV collagen is a major structural component of the renal basement membrane and plays a critical role in glomerular filtration. Its C-terminal noncollagenous domain is referred to as the NC1 domain. In indirect immunohistochemical staining of frozen sections, a mouse-derived monoclonal anti-NC1 antibody (12D) stains basement membranes in injured kidneys but does not stain those in normal kidneys. In contrast, a rabbit-derived polyclonal anti-NC1 antiserum (polyclonal NC1 antibodies) stains basement membranes in both injured and normal kidneys. In the present study, to clarify the distinctive characteristics of 12D, we investigated differences between 12D and other anti-NC1 antibodies, including analyses involving nephritic urine.

Methods

Sodium dodecyl sulfate polyacrylamide gel electrophoresis (SDS-PAGE) and Western blotting (WB) were performed. NC1 treated with the reducing agent 2-mercaptoethanol (2ME) (reduced NC1) and NC1 not treated with 2ME (non-reduced NC1) were subjected to electrophoresis, and their reactivity with 12D, nephritic urine, polyclonal NC1 antibodies (polyclonal NC1 antibodies), and an anti-yp08 polyclonal antiserum (yp08 antibodies) was analyzed by WB. The antigen for the anti-yp08 antibody was a synthetic peptide, yp08 (PFISR CAVCE APAVV IAVHS), corresponding to an amino acid sequence within the NC1 domain of the type IV collagen α5 chain.

Results

12D reacted with non-reduced NC1 but did not react with reduced NC1, in which disulfide bonds had been cleaved by 2ME. The two polyclonal antibodies (polyclonal NC1 antibodies and yp08 antibodies) reacted with both non-reduced NC1 and reduced NC1. Among 23 nephritic urine samples, 13 reacted with non-reduced NC1, whereas 20 samples, including these 13, reacted with reduced NC1.

Conclusion

Unlike polyclonal NC1 antibodies or factors present in nephritic urine, 12D requires intact disulfide bonds for its interaction with NC1. These results demonstrate that the binding of 12D to NC1 is disulfide bond-dependent. Because disulfide bonds are formed through oxidative processes, these findings suggest that kidneys stained by 12D in indirect immunohistochemistry are in an oxidized state.

## Introduction

The aim of this study was to characterize the binding properties of the monoclonal anti-NC1 antibody 12D in comparison with polyclonal antibodies and nephritic urine, with particular focus on the role of disulfide bonds in NC1 and the mechanisms underlying its selective staining of injured kidneys.

Type IV collagen is a major component of basement membranes in biological tissues. It consists of six α chains, three of which assemble to form a single molecule. The possible triple-chain compositions are α1α2α1 (α121), α345, and α565. The α121 network is widely distributed throughout the body, including the kidney, whereas α345 is abundant in glomeruli, α565 is localized mainly in renal tubules and Bowman’s capsule, and α121 is present in the mesangial region [1-3].

Type IV collagen is composed of three distinct domains: the N-terminal 7S domain, the central triple-helical (TH) domain, and the C-terminal noncollagenous 1 (NC1) domain. In vivo, NC1 forms trimers composed of three α chains, and two trimers associate to form a hexamer [4].

The authors previously extracted NC1 from bovine renal cortex using collagenase [5-7] and used it as an antigen to measure anti-NC1 antibodies in serum and urine by enzyme-linked immunosorbent assay (ELISA). They reported that anti-NC1 antibodies are occasionally present in the blood of healthy individuals and are detected in the urine of many patients with glomerulonephritis, irrespective of disease subtype [8-10].

Furthermore, a monoclonal anti-NC1 antibody produced by the authors (mouse-derived, 12D) exhibited a unique staining pattern. When applied to frozen kidney sections by indirect immunohistochemistry, 12D stained basement membranes in monkey anti-glomerular basement membrane (GBM) antibody nephritis and in human glomerulonephritis, but did not stain those of normal kidneys [11]. Notably, this selective staining was observed regardless of the type of human glomerulonephritis.

In contrast to conventional immunohistochemical detection using anti-human IgG antibodies, which identifies immune complex deposition, 12D directly reacts with NC1 and selectively detects injured renal tissue. Consistently, anti-NC1 antibodies are detectable in the urine in association with renal injury.

These findings suggest that NC1 undergoes structural or conformational alterations specifically in injured renal basement membranes, leading to the exposure of antigenic sites that are not accessible in normal tissues. However, the molecular basis for this selective recognition, including the role of disulfide bonds and structural integrity of NC1, remains unclear.

In human glomerulonephritis, the staining sites of 12D include the basement membranes of glomeruli, renal tubules, and Bowman’s capsule. The α chain common to these basement membranes is the α5 chain. Tokura et al. have shown through immunohistochemistry that the distribution of α chains is strictly differentiated in the renal basement membrane, with α5 chains being limited to the glomerular basement membrane, Bouman's capsule basement membrane, and a portion of the renal tubules [12].

If α5-chain NC1 serves as the antigen inducing the production of anti-NC1 antibodies excreted in urine, it is essential to identify which regions of α5-chain NC1 are involved. Therefore, the amino acid sequence of α5-chain NC1 was synthesized as overlapping 20-residue peptides, and their reactivity with nephritic urine was examined by ELISA. Nephritic urine showed strong reactivity with multiple peptides, including yp08 [13].

In the present study, we examined differences between 12D and nephritic urine, as well as self-produced polyclonal antibodies (polyclonal anti-NC1 antibodies and anti-yp08 antibodies), by Western blotting (WB), and investigated why 12D selectively stains injured kidneys in indirect immunohistochemistry.

## Materials and methods

Overview of NC1 preparation, SDS-PAGE, and Western blotting

NC1 was extracted from bovine renal cortical basement membranes by collagenase digestion, as previously described [5-7]. NC1 samples were analyzed by sodium dodecyl sulfate polyacrylamide gel electrophoresis (SDS-PAGE) according to the method of Laemmli [14], followed by WB.

For SDS-PAGE, NC1 treated with the reducing agent 2-mercaptoethanol (2ME) (reduced NC1) and NC1 not treated with 2ME (non-reduced NC1) were used. NC1 (2-4 µg per lane, corresponding to 30 µg per gel) was electrophoresed on 7.5-12% SDS-PAGE gels. Proteins were transferred to polyvinylidene fluoride (PVDF) membranes (Millipore) using a semi-dry transfer apparatus.

**Figure 1:**
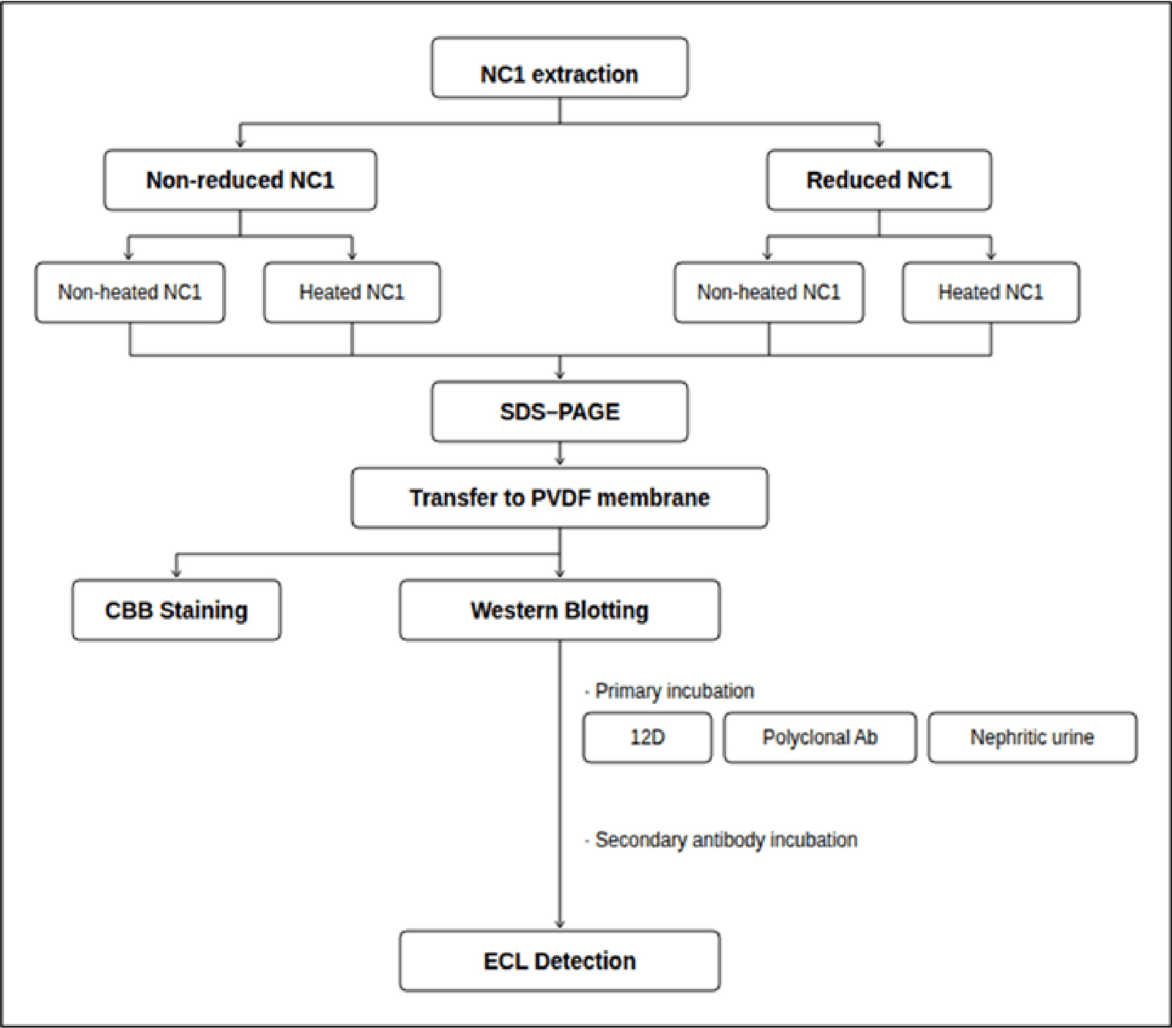
Diagram of method. The details of the figure are provided in the main text. SDS-PAGE: sodium dodecyl sulfate polyacrylamide gel electrophoresis; PVDF: polyvinylidene fluoride; CBB: Coomassie Brilliant Blue; ECL: enhanced chemiluminescence; NC1: type IV collagen NC1 domain; Reduced: reduced by 2-mercaptoethanol; Heated: 95℃ for 15 minutes.

After transfer, membranes were air-dried, cut into strips, immersed in sample solutions, and incubated at room temperature for one to two hours, followed by washing. Enzyme-labeled secondary antibodies were then added and incubated at room temperature for one hour, followed by washing. Membranes were subsequently immersed in enhanced chemiluminescence (ECL) Western blot detection reagent (Amersham™, Cytiva, Marlborough, MA), and chemiluminescent signals were detected by exposure to photographic film and development (Figure 1).

Samples

A mouse-derived monoclonal anti-NC1 antibody (12D) and rabbit-derived polyclonal antisera (polyclonal NC1 antibodies raised against NC1 derived from bovine renal cortex, and yp08 antibodies raised against the synthetic peptide yp08) were produced by the authors.

Nephritic urine samples used in this study were provided in 2003 by the National Hospital Organization Chiba-East Hospital together with health screening data, after obtaining informed consent from all subjects and anonymization of the samples.

Urine samples from healthy individuals, used as controls in ELISA measurements, were provided in 2018 by volunteers at Tokyo Dental College Suidobashi Hospital after anonymization [15]. No urinary protein, glucose, or occult blood was detected in the urine from the volunteers. Stored frozen urine samples were analyzed in 2023.

Experimental procedures

Effect of Reduction and Heating on NC1

First, the effect of heating on the electrophoretic behavior of NC1 was examined. Non-reduced NC1 and reduced NC1 samples were prepared and either heated at 95°C for 15 minutes or left unheated prior to electrophoresis. The samples were separated by 10% SDS-PAGE and transferred to membrane filters. Membranes were stained with Coomassie Brilliant Blue (CBB). NC1 was loaded at 2 µg or 4 µg per lane.

Reactivity of 12D With NC1 Under Different Reduction and Heating Conditions

Non-reduced and reduced NC1 samples were either heated at 95°C for 15 minutes or left unheated prior to separation by 10% SDS-PAGE. After electrophoresis and transfer to membranes, differences in reactivity between NC1 and 12D were analyzed by WB.

The monoclonal antibody 12D was diluted 1:500-1:1,000 in phosphate-buffered saline (PBS). An enzyme-conjugated anti-mouse IgG antibody (Bethyl Laboratories, Montgomery, TX), diluted 1:10,000, was used as the secondary antibody.

Reactivity of Polyclonal Antibodies With NC1 Under Different Reduction and Heating Conditions

Non-reduced and reduced NC1 samples were either heated at 95°C for 15 minutes or left unheated prior to separation by 10% SDS-PAGE. Following electrophoresis and transfer to membranes, differences in reactivity between NC1 and polyclonal antibodies were examined by WB.

The polyclonal antibodies used were polyclonal NC1 antibodies (1:2,000), affinity-purified anti-NC1 polyclonal antibody (anti-NC1ap; 1:2,000), yp08 antibodies (1:250-1:500), and affinity-purified anti-yp08 antibody (anti-yp08AF; 1:250-1:500). An enzyme-conjugated anti-rabbit IgG antibody (Bethyl Laboratories), diluted 1:10,000, was used as the secondary antibody.

Western Blotting of NC1 With Nephritic Urine Under Reducing and Non-reducing Conditions

To compare the results with previously obtained ELISA data from nephritic urine, the reactivity of nephritic urine with non-reduced NC1 and with reduced NC1 was examined by WB. The reduced NC1 used in this experiment was heated prior to electrophoresis. Enzyme-labeled anti-human IgG antibodies (1: 20,000) were used for detection.

ELISA was performed as follows: NC1 was immobilized on 96-well microplates, and nephritic urine samples diluted fivefold with PBS (pH 7.4) (N = 23) were added. Urinary anti-NC1 antibodies were detected using enzyme-labeled anti-human IgG antibodies (40,000). Positivity was defined as values exceeding the mean + 3 standard deviation (SD) of healthy control urine samples (N = 19) [15].

Effect of Heating on NC1 Reactivity With Nephritic Urine

Using three nephritic urine samples that were positive by both ELISA and WB, WB was performed to compare reactivity with non-reduced NC1 and reduced NC1 under heated and non-heated conditions.

## Results

Summary of results

We first present a summary of the measurement results in Table 1.

**Table 1:** Summary of results. The details of the table are provided in the main text. CBB: Coomassie Brilliant Blue; Polyclonal: polyclonal NC1 antibodies, yp08 antibodies; N/A: no execution.

		Non-reduced NC1		Reduced NC1	
		Non-heated	Heated	Non-heated	Heated
CBB		〇	〇	〇	〇
12D		〇	〇	×	×
Polyclonal antibodies		〇	〇	〇	〇
Nephritic urine		〇	N/A	N/A	〇

Effect of reduction and heating on NC1

**Figure 2:**
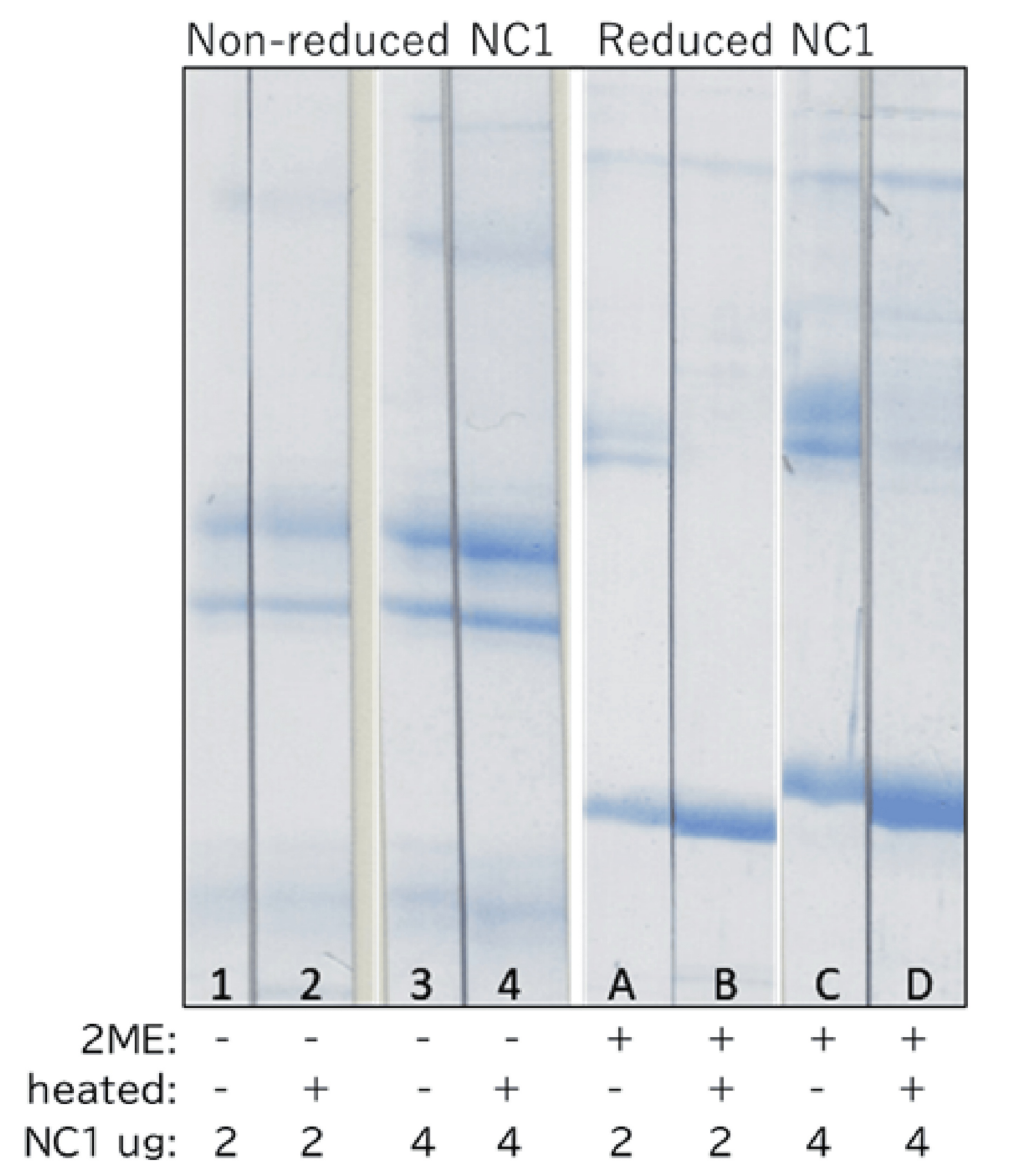
Coomassie Brilliant Blue (CBB) staining of NC1 under reducing and non-reducing conditions, with or without heating. NC1 samples were treated with or without 2-mercaptoethanol (2ME) to generate reduced (2ME +) or non-reduced (2ME −) conditions and were either heated (+) or left unheated (−) prior to SDS-PAGE. NC1 was loaded at 2 μg or 4 μg per lane. The intensity of CBB staining increased proportionally with the amount of NC1 applied. SDS-PAGE: sodium dodecyl sulfate polyacrylamide gel electrophoresis.

In SDS-PAGE, non-reduced NC1 showed two dimer bands that were more intensely stained by CBB than the monomer bands, regardless of heating. In contrast, reduced NC1 exhibited stronger staining of monomer bands than non-reduced NC1 (Figure 2).

In reduced NC1 without heating, two dimer bands remained despite treatment with 2ME. However, upon heating, these dimer bands became almost undetectable, and monomer staining was markedly enhanced.

In reduced NC1, the temperature and duration of heating modulated the effective reducing strength of 2ME. Accordingly, the dimer bands persisting in non-heated, reduced NC1 were interpreted as species in which disulfide bonds had been cleaved, but intermolecular linkages remained. These residual dimers were presumed to represent sulfilimine bonds, as previously reported [16].

Reactivity of 12D under different reduction and heating conditions

**Figure 3:**
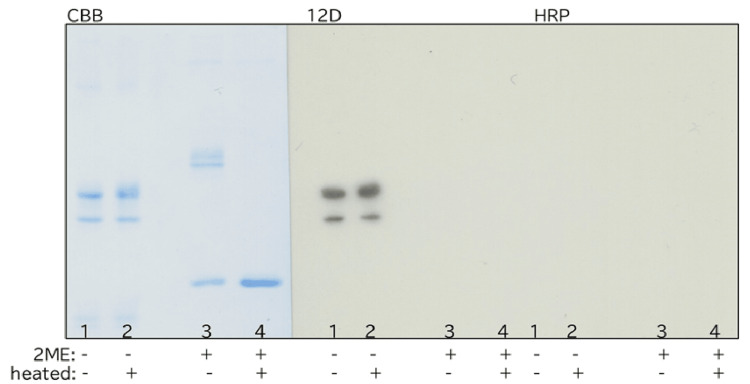
(A) Western blot analysis of NC1 reacted with the monoclonal antibody 12D under reducing and non-reducing conditions, with or without heating. Reduction was performed with 2-mercaptoethanol (2ME), and heating was either applied or omitted prior to SDS-PAGE. Lane assignments were as follows: lane 1, non-reduced NC1 without heating; lane 2, non-reduced NC1 with heating; lane 3, reduced NC1 without heating; and lane 4, reduced NC1 with heating. Following SDS-PAGE, NC1 was analyzed by Western blotting using the monoclonal antibody 12D. Reduction with 2ME markedly affected the reactivity between 12D and NC1, whereas heating had no detectable effect. SDS-PAGE: sodium dodecyl sulfate polyacrylamide gel electrophoresis; CBB: Coomassie Brilliant Blue; HRP: horseradish peroxidase.

**Figure 4:**
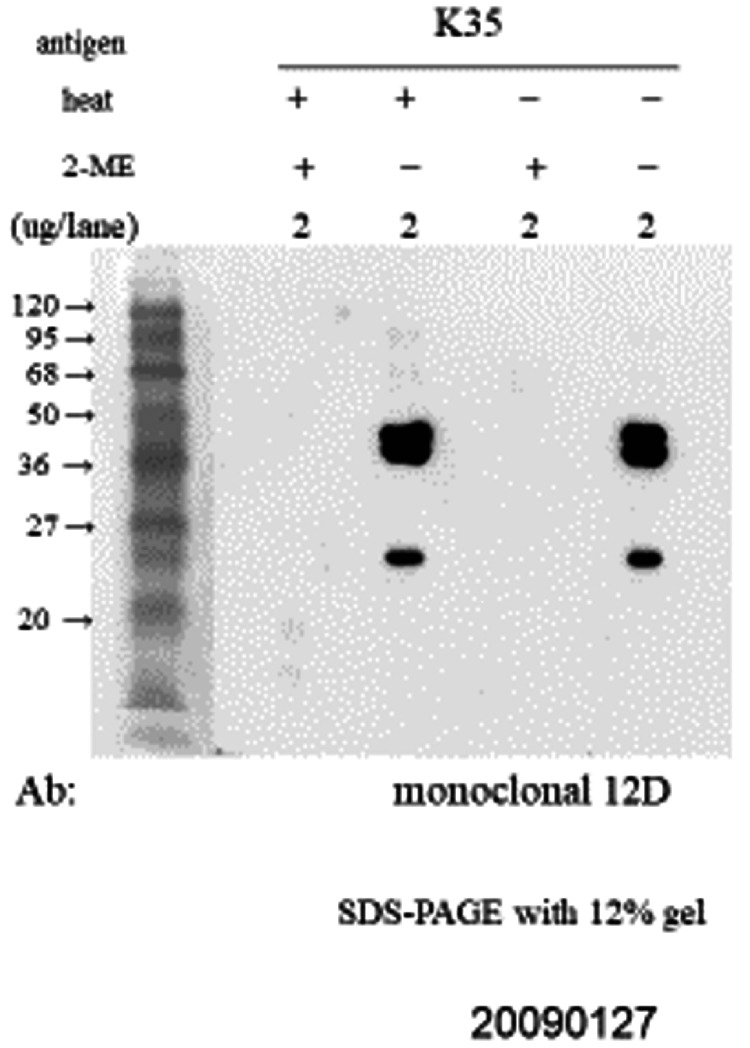
(B) Western blot analysis of the monoclonal antibody 12D reacted with NC1 (K35). This experiment was performed to examine whether heating affects the interaction between the monoclonal antibody 12D and NC1. Both non-reduced NC1 and reduced NC1 were subjected to heating or non-heating conditions prior to SDS-PAGE and subsequent Western blot analysis using monoclonal antibody 12D. Reduced NC1 treated with 2-mercaptoethanol (2ME) or non-reduced NC1 without 2ME was loaded at 2 μg per lane onto a 12% polyacrylamide gel and electrophoresed at 100 V for approximately 80 minutes. Prestained protein molecular weight markers (Broad Range, Nacalai Tesque, Kyoto, Japan) were used. After electrophoresis, gels were stained with CBB Stain One (Nacalai Tesque) to visualize separated proteins. Gel images were recorded using a Printgraph gel documentation system (AE-6932GXCF, ATTO Corporation, Tokyo, Japan). No difference was observed in the reactivity of monoclonal antibody 12D with non-reduced NC1, regardless of the presence or absence of heating. SDS-PAGE: sodium dodecyl sulfate polyacrylamide gel electrophoresis; CBB: Coomassie Brilliant Blue.

12D reacted with non-reduced NC1 but did not react with reduced NC1. Although 12D reacted with non-reduced NC1 dimers regardless of heating, it did not react with dimers present in reduced NC1 without heating (Figures 3, 4). Thus, the presence of a dimeric form alone was not sufficient for reactivity between 12D and NC1.

The epitope recognized by 12D on NC1 was stable to heating but sensitive to reduction by 2ME, indicating that the epitope was lost upon disruption of disulfide bonds.

Reactivity of polyclonal antibodies under different reduction and heating conditions

**Figure 5:**
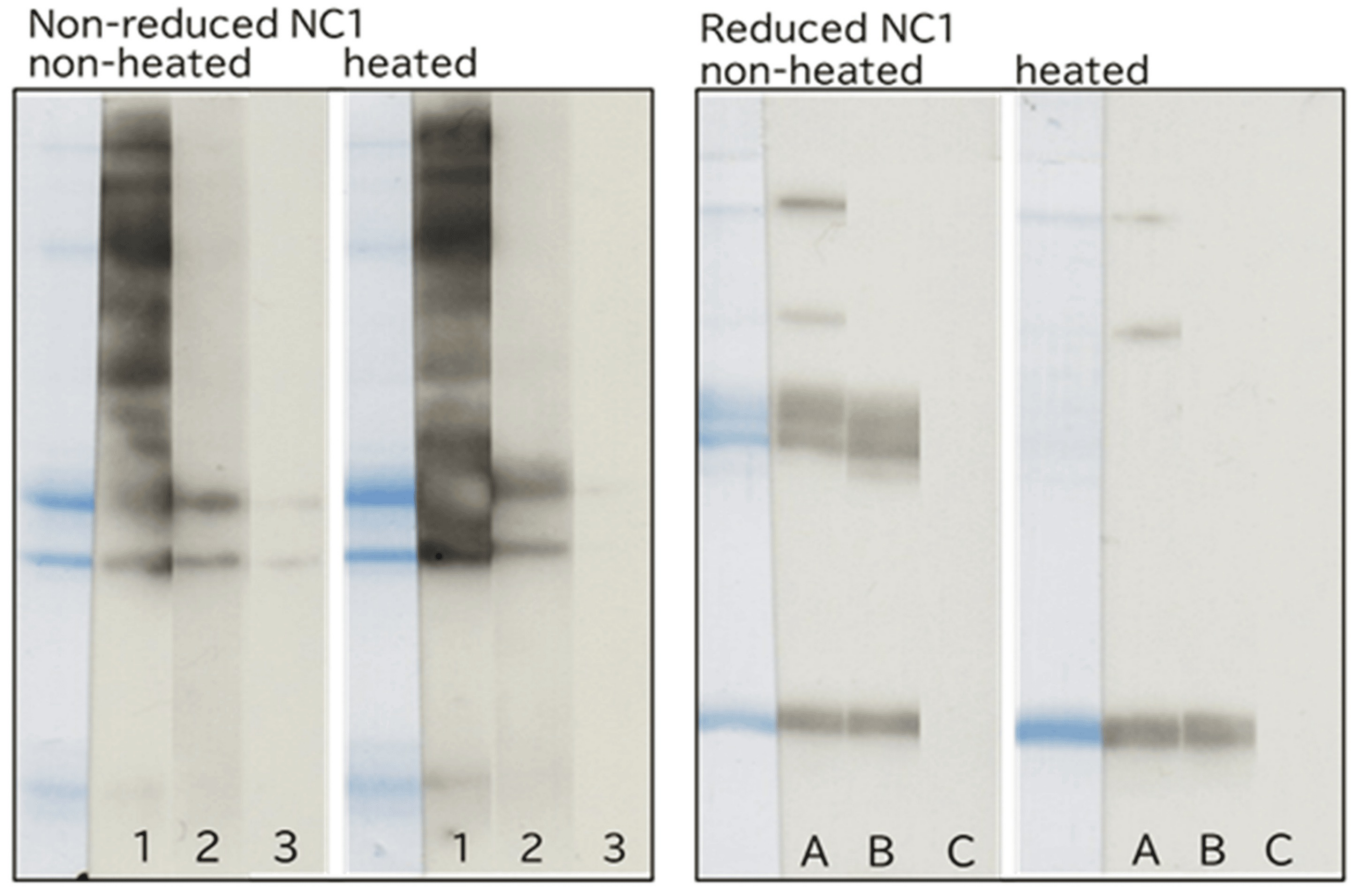
(A) Western blot analysis of NC1 reacted with polyclonal antibodies under reducing and non-reducing conditions, with or without heating. Lane 1 (A) shows reactivity with polyclonal anti-NC1 antibodies, lane 2 (B) shows reactivity with anti-yp08 antibodies, and lane 3 (C) represents a blank control incubated with enzyme-labeled secondary antibody only. Both polyclonal anti-NC1 antibodies and anti-yp08 antibodies reacted with NC1 regardless of reduction or heating, indicating that these antibodies recognize epitopes resistant to changes induced by reduction or heat treatment.

**Figure 6:**
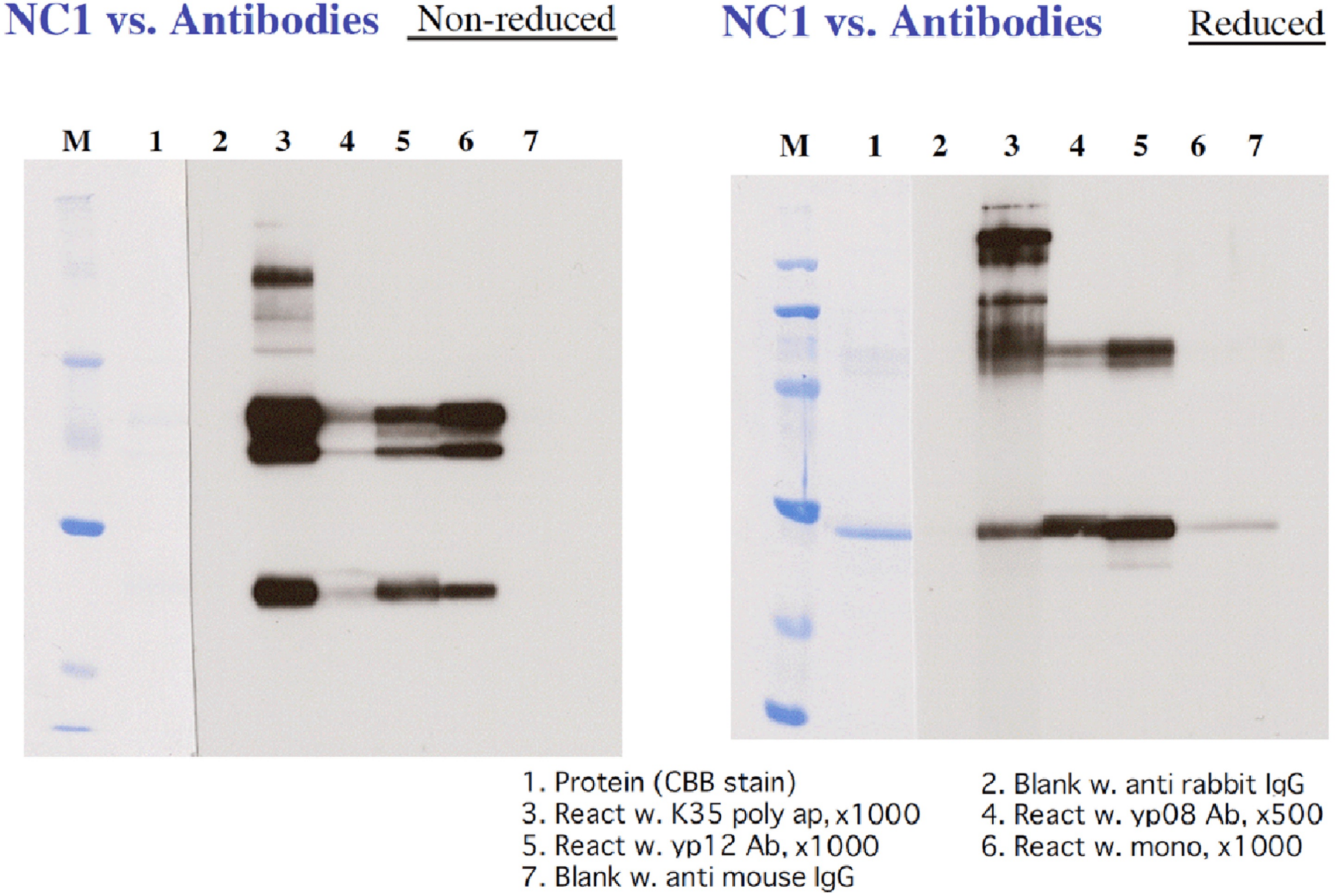
(B) Western blot analysis of antibodies reacted with NC1 (100823). Lane 3 (K35ap) represents affinity-purified anti-NC1 polyclonal antibodies. Lane 5 (yp12Ab) represents antiserum raised against the synthetic peptide yp12 derived from the NC1 domain of the α5 (IV) collagen chain; the yp12 sequence is also present in the NC1 domain of the α1 (IV) collagen chain. Lane 6 (mono) represents the monoclonal antibody 12D. CBB: Coomassie Brilliant Blue.

Polyclonal antibodies (polyclonal NC1 antibodies and yp08 antibodies) reacted with both non-reduced NC1 and reduced NC1 regardless of heating (Figures 5, 6). In this respect, the reactivity profile of polyclonal antibodies clearly differed from that of 12D, which failed to react with reduced NC1.

Polyclonal antibodies reacted with dimers remaining in reduced NC1 without heating, as well as with monomers generated by reduction. The observation that yp08 antibodies reacted with dimers of reduced NC1 without heating indicates that NC1 derived from the α5 chain was retained within these dimers. Furthermore, upon heat-assisted reduction, dimeric NC1 species were eliminated and converted into monomeric forms, which remained strongly reactive with yp08 antibodies. These findings indicate that α5-chain-derived NC1 is preserved within the monomeric fraction following complete disruption of intermolecular linkages.

Western blotting of NC1 with nephritic urine

**Figure 7:**
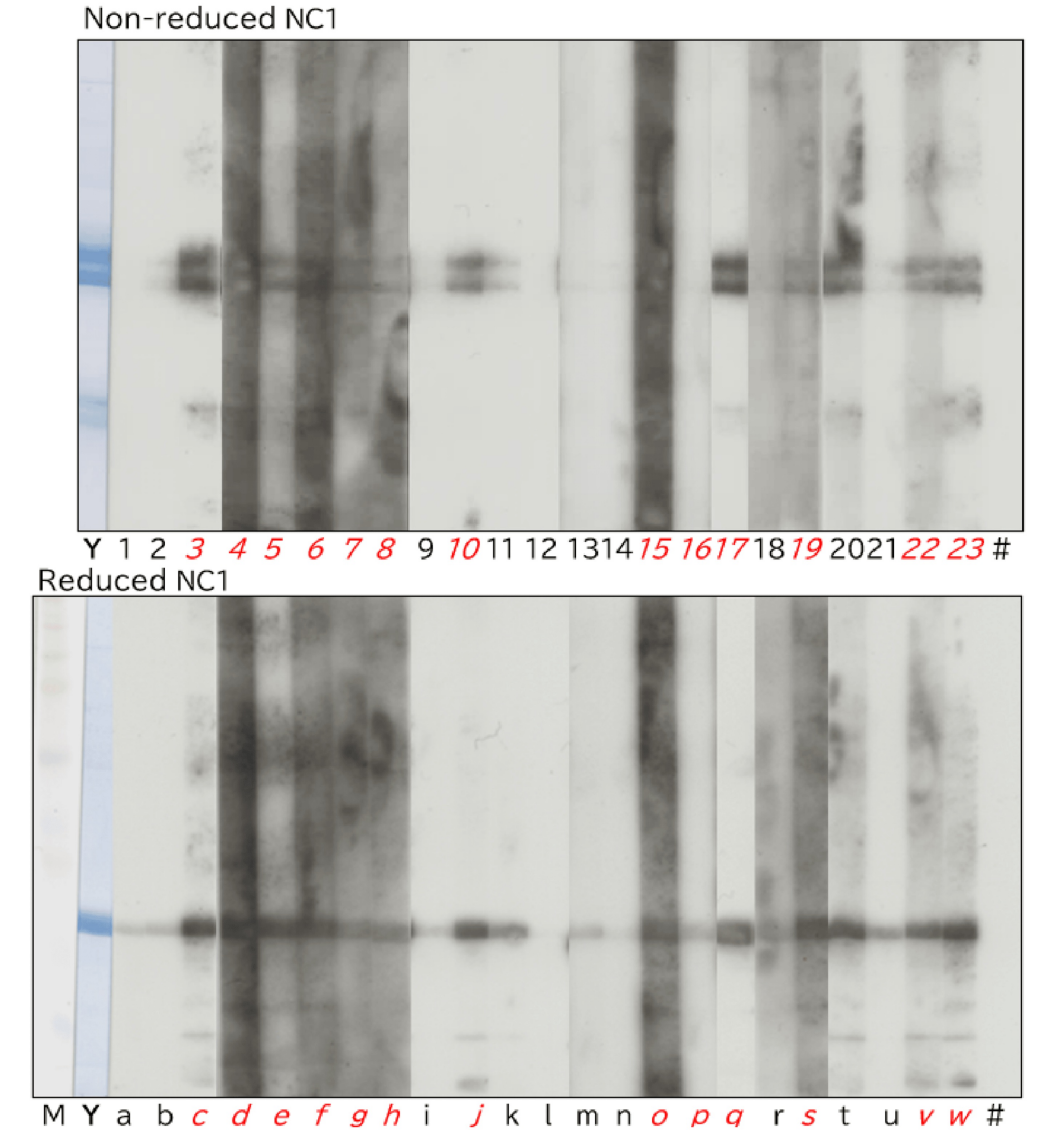
Western blot analysis of NC1 reacted with urine samples from patients with glomerulonephritis under reducing and non-reducing conditions. For non-reduced NC1, lane Y shows CBB staining, lane Z shows reactivity with polyclonal anti-NC1 antibodies, lane X represents a blank control incubated with enzyme-labeled secondary antibody only, and lane # indicates a spacer. Lanes 1-23 correspond to urine samples from patients with glomerulonephritis. Sample numbers shown in italics indicate ELISA-positive cases, defined as values exceeding the mean + 3 SD of healthy controls. For reduced NC1, lane M indicates the molecular weight marker. CBB: Coomassie Brilliant Blue; ELISA: enzyme-linked immunosorbent assay.

Compared with ELISA, WB detected positive reactions more clearly (Figure 7). When positivity was defined in ELISA as values exceeding the mean + 3 SD of healthy urine controls, 13 of 23 nephritic urine samples were positive. Although ELISA allows analysis of a large number of samples, the results can be influenced by variations in control samples, microplates, and blocking conditions.

WB analysis showed that 13 of the 23 samples reacted with the non-reduced NC1 dimers. Among these, two samples (lanes 11 and 20) were negative by ELISA. Of the remaining 10 samples, six (lanes 1, 12, 13, 14, 16, and 18) showed no reactivity, three (lanes 2, 9, and 21) showed only weak reactivity with the NC1 dimers, and one sample (lane 15) exhibited an uninterpretable reaction pattern due to nonspecific adhesion of urinary components to the membrane. Among these 10 samples, all except two (lanes 15 and 16) were negative by ELISA.

When the reduced NC1 monomers were used, 20 of the 23 samples showed reactivity, including all 13 samples that reacted with the non-reduced NC1 dimers. Of the remaining three samples, one (lane 1) showed no reactivity; this sample corresponded to the same sample that was negative for the non-reduced NC1 dimers (lane 12). And the other two samples (lanes i and n) showed only weak reactivity with the NC1 monomers (Table 2).

**Table 2:** Measurement results for each urine sample. The details of the table are provided in the main text. ELISA: enzyme-linked immunosorbent assay; 2ME: 2-mercaptoethanol; WB: Western blotting; Non-reduced: non-reduced NC1 for WB; Reduced: reduced NC1 for WB; WB 2ME-: WB without 2ME; WB 2ME+: WB with 2ME; 〇: reactivity; X: no reactivity; △: little reactivity; N/A: no decision.

Non-reduced		1	2	3	4	5	6	7	8	9	10	11	12	13	14	15	16	17	18	19	20	21	22	23
Reduced		a	b	c	d	e	f	g	h	i	j	k	l	m	n	o	p	q	r	s	t	u	v	w
ELISA		×	×	〇	〇	〇	〇	〇	〇	×	〇	×	×	×	×	〇	〇	〇	×	〇	×	×	〇	〇
WB 2ME-		×	△	〇	〇	〇	〇	〇	〇	△	〇	〇	×	×	×	N/A	×	〇	×	〇	〇	△	〇	〇
WB 2ME+		〇	〇	〇	〇	〇	〇	〇	〇	△	〇	〇	×	〇	△	〇	〇	〇	〇	〇	〇	〇	〇	〇

Comparison of nephritic urine reactivity in ELISA and WB

**Figure 8:**
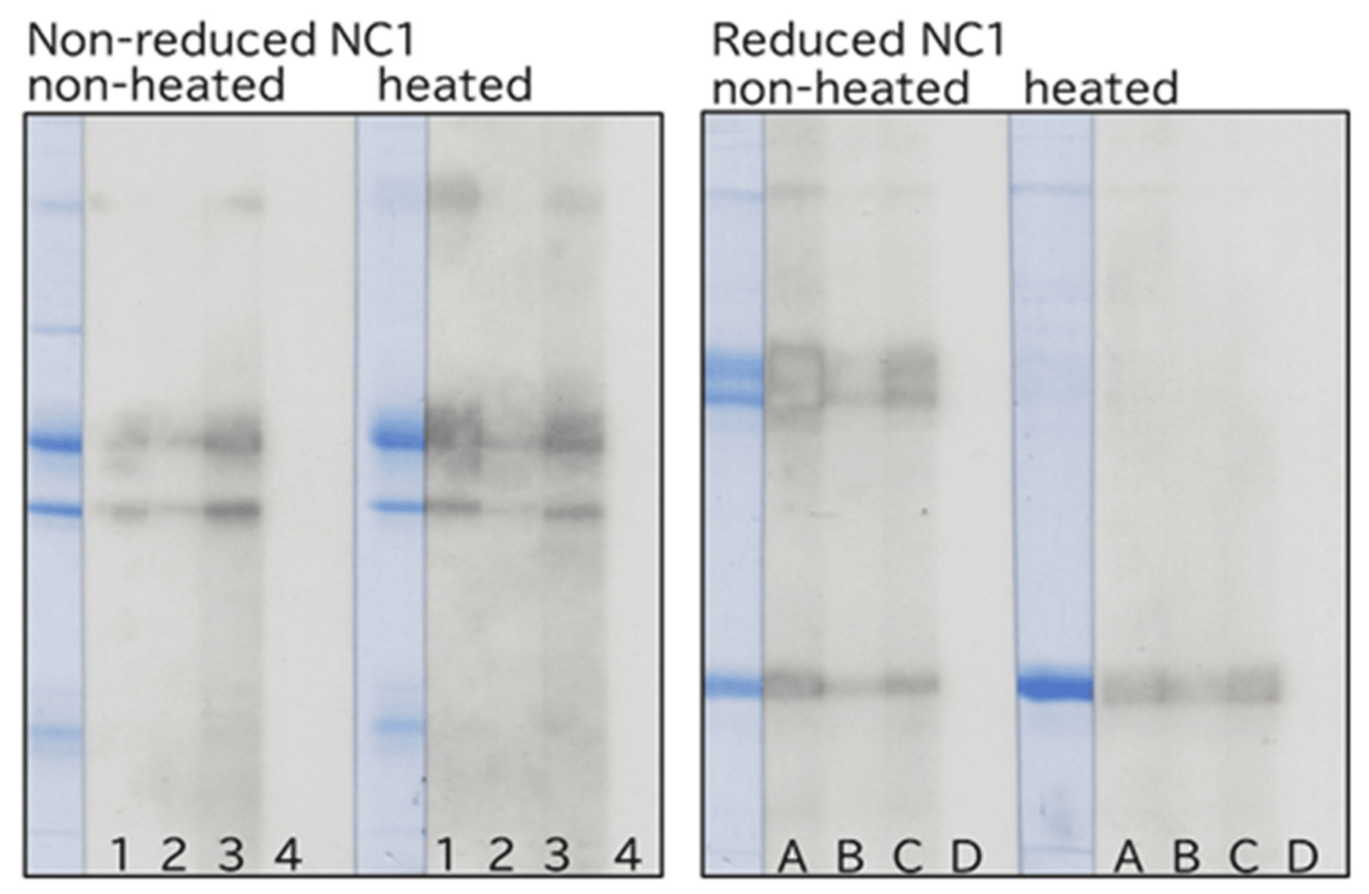
Western blot analysis of NC1 reacted with urine samples from patients with glomerulonephritis under reducing and non-reducing conditions, with or without heating. Lane 1 (A) corresponds to sample 3c, lane 2 (B) to sample 10j, lane 3 (C) to sample 17q, and lane 4 (D) represents a blank control. All urine samples analyzed in this figure were positive by ELISA. ELISA: enzyme-linked immunosorbent assay.

Three nephritic urine samples that reacted with NC1 in ELISA and showed reactivity with NC1 in WB regardless of reduction were further examined (Figure 8). Similar to polyclonal antibodies, these urine samples reacted with NC1 in WB irrespective of reduction or heating conditions.

## Discussion

Characteristics of extracted NC1

Extracted NC1 may differ among preparations depending on the breed, age, and sex of the source bovine kidneys, as well as alterations occurring during collagenase extraction and storage. Consequently, extracted NC1 may exist in hexameric forms that differ from those present in vivo. Furthermore, during SDS-PAGE, sodium dodecyl sulfate dissociates NC1 hexamers, resulting in separation into bands according to molecular weight.

Differences in reactivity with various anti-NC1 antibodies were observed between the NC1 used in previous experiments and the NC1 used in the present study. In earlier Western blotting experiments, monomers were present in non-reduced NC1, and 12D reacted with these monomers (Figure 4). In addition, one of the two dimer bands observed in non-reduced NC1 (with a weak third band also detected) persisted as a dimer after reduction, and various polyclonal antibodies reacted with this species (Figure 6). Across all NC1 preparations, these polyclonal antibodies, unlike 12D, reacted with reduced NC1 in Western blotting, indicating that disulfide bonds are not strictly required for their interaction with NC1.

Presence of autoantibodies in nephritic urine

The authors previously demonstrated by ELISA that anti-NC1 antibodies are elevated in the urine of patients with nephritis, and further demonstrated the presence of urinary anti-NC1 antibodies by Western blotting using reduced NC1 and, in a limited number of cases, non-reduced NC1 [7-9].

In the present study, Western blotting revealed that in many nephritic urine samples, anti-NC1 antibodies reacted not only with reduced NC1 monomers but also with non-reduced NC1 dimers (Figures 7, 8). In the rare disease of anti-GBM antibody nephritis, autoantibodies are detected in serum; however, in most forms of nephritis, autoantibodies generated against NC1 at sites of renal injury are detected predominantly in urine rather than in blood.

The absence of detectable anti-NC1 antibodies in serum may be due to degradation of these antibodies after entering the circulation, or to the possibility that, as nephritis progresses, anti-NC1 antibodies are directly excreted into urine.

Monoclonal anti-NC1 antibody (12D) and immunohistochemistry

**Figure 9:**
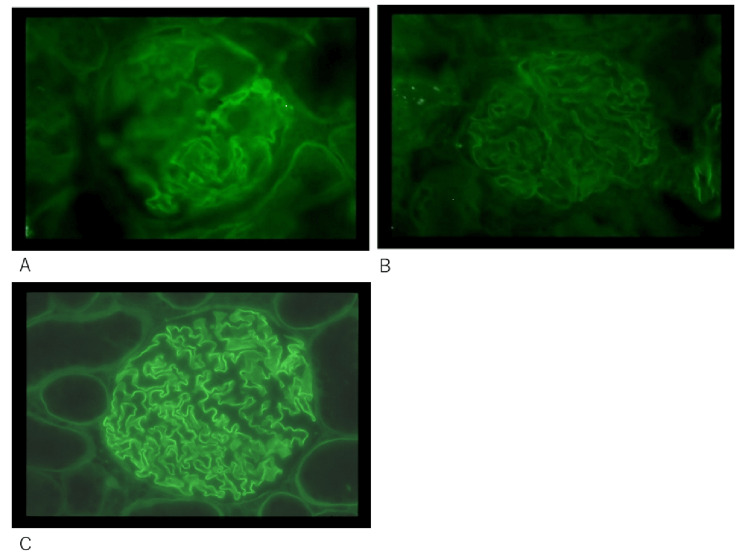
Immunohistochemical staining using the monoclonal antibody 12D. (A) Indirect immunohistochemical staining of NC1 using the monoclonal antibody 12D. (B) Direct immunofluorescence staining using a fluorescently labeled anti-mouse IgG secondary antibody as a blank control. (C) Direct immunohistochemical staining using an enzyme-labeled anti-monkey IgG antibody. Frozen kidney sections were obtained from a monkey glomerular basement membrane nephritis model (No. 2301) six weeks after NC1 administration. Panels A and B represent consecutive frozen sections. In panel A, the monoclonal antibody 12D strongly reacted with a portion of NC1. In panel B, the fluorescently labeled anti-mouse IgG secondary antibody showed weak cross-reactivity with monkey IgG. In contrast, in panel C, the enzyme-labeled anti-monkey IgG antibody strongly reacted with deposited monkey IgG.

In indirect immunohistochemical staining of frozen sections, 12D was the first monoclonal antibody reported to distinguish between normal and injured kidneys (Figure 9) [11].

In contrast, rabbit-derived polyclonal anti-NC1 antibodies stain normal kidneys as well [11]. Because NC1 is similarly exposed in frozen sections of both normal and injured kidneys, the authors concluded that alterations occur in injured kidneys that are not present in normal kidneys [11,13].

Consistent with this interpretation, polyclonal anti-NC1 antibodies that stain normal kidneys reacted with NC1 in Western blotting regardless of reduction (Figure 5). In contrast, 12D, which does not stain normal kidneys, reacted with non-reduced NC1 but not with reduced NC1 (Figures 3, 4, 6).

The epitope of NC1 recognized by 12D exists in non-reduced NC1 and is lost upon reduction by 2ME, suggesting that this epitope depends on a structure involving reducible disulfide bonds. Supporting the importance of disulfide bonds in antigenicity, a cryptic disulfide bond-dependent epitope within α3(IV)NC1 has been identified as the target antigen in Goodpasture syndrome [17]. In addition, cleavage of disulfide bonds within the antigenic region of the C-type lectin-like domain (CTLD) of the M-type phospholipase A2 receptor (PLA2R) results in loss of antigenicity in membranous nephropathy [18]. Together, these findings underscore the critical role of disulfide bonds in maintaining disease-relevant antigenic structures.

**Figure 10:**
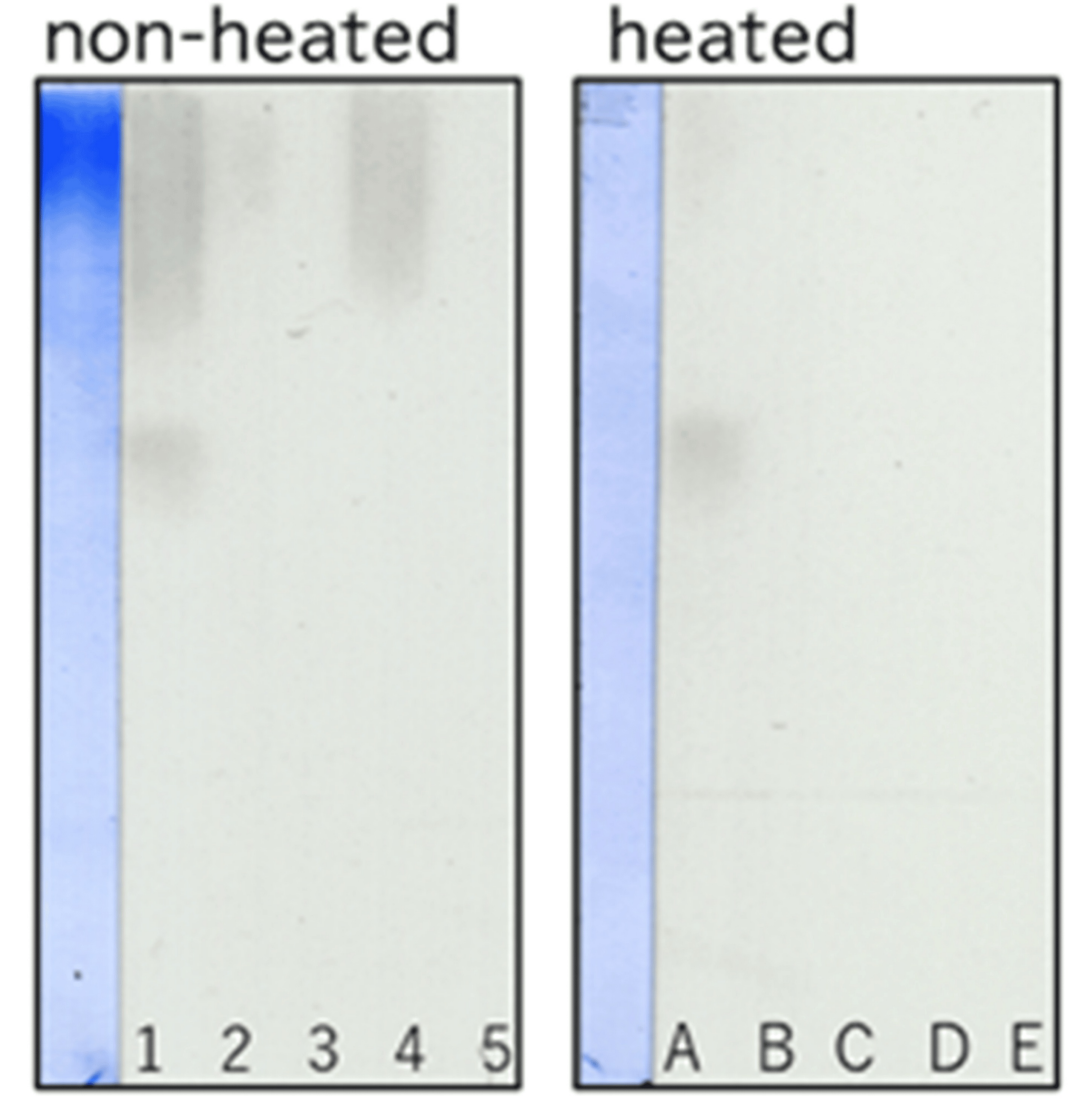
Native PAGE analysis of NC1 and antibody reactivity. NC1 extracted and purified from normal bovine kidney cortex was subjected to 7.5% native PAGE, followed by Western blot analysis using antibodies. Lane 1 (a) shows reactivity with polyclonal anti-NC1 antibodies, lane 2 (b) shows reactivity with anti-yp08 antibodies, lane 3 (c) shows PBS-T as a blank control, lane 4 (d) shows reactivity with the monoclonal antibody 12D, and lane 5 (e) shows PBS-T as a blank control. Heating caused the disappearance of the upper band observed in non-heated NC1 and resulted in the appearance of lower-molecular-weight bands. Both polyclonal anti-NC1 antibodies and the monoclonal antibody 12D reacted with the upper band of non-reduced NC1, whereas anti-yp08 antibodies showed only weak reactivity. PAGE: polyacrylamide gel electrophoresis; PBS-T: phosphate-buffered saline with Tween 20.

Abnormal protein oxidation contributes to the pathogenesis of various diseases [19], and proteins contain oxidation-prone amino acids such as methionine, rendering them susceptible to oxidative modification [20]. NC1 analyzed by Western blotting after native polyacrylamide gel electrophoresis (PAGE), which preserves higher-order protein structure, reacted with 12D despite being extracted from normal kidneys (Figure 10), suggesting that extracted NC1 differs structurally from NC1 in vivo.

The α5-chain NC1 region stained by 12D in immunohistochemistry contains a total of 21 oxidation-prone amino acids, including nine methionine and 12 cysteine residues [13]. Other α-chain NC1 domains similarly contain numerous methionine and cysteine residues. Oxidative conditions readily promote disulfide bond formation [21,22]. In injured kidneys, oxidation-prone amino acids within the same α chain or between different α chains may form novel disulfide bonds, thereby stabilizing NC1 in a conformation distinct from that in normal tissue.

Kalluri et al. reported that anti-GBM antibodies from patients with Goodpasture syndrome do not stain normal kidneys but do stain kidneys rendered injured by treatment with reactive oxygen species (ROS) [23]. Similarly, these antibodies failed to stain normal rat kidneys but stained rat kidneys perfused with hydrogen peroxide (H₂O₂). If the antigen were merely a cryptic epitope masked beneath the tissue surface, it would be expected to be exposed in frozen sections of normal kidneys. Therefore, ROS or H₂O₂ treatment is thought not simply to expose cryptic epitopes, but rather to generate antigenic structures under oxidative conditions, with treated kidneys presumed to be in an oxidized state.

In indirect immunohistochemistry, sheep-derived anti-rat GBM polyclonal antibodies used by Kalluri et al. stained both normal and H₂O₂-treated rat kidneys [23]. The relationship between patient-derived anti-GBM antibodies and sheep-derived anti-rat GBM polyclonal antibodies described by Kalluri et al. is analogous to the relationship between 12D and rabbit-derived polyclonal anti-NC1 antibodies described in the present study. Both anti-GBM antibodies from patients with Goodpasture syndrome and the mouse-derived monoclonal antibody 12D are therefore considered to selectively stain basement membranes of kidneys in an oxidized state.

In this study, we used Western blotting to examine the differences between 12D and other antibodies, including those present in the urine of nephritic models, and to discuss the characteristics of 12D inferred from these comparisons. However, we were unable to determine the structural state of disulfide bonds in injured kidneys, and our discussion relied solely on Western blotting. This inevitably renders the interpretation somewhat one‑sided. To achieve a more multifaceted analysis, it remains an important task to identify additional methodological approaches that can substantiate the points under discussion.

## Conclusions

Glomerulonephritis is a common renal disease for which effective therapeutic and preventive strategies remain limited. Over the past 25 years, the authors have pursued the development of experimental approaches to facilitate research on glomerulonephritis. During this period, some findings were presented at scientific meetings, some were published in Japanese journals, and others remained unpublished despite experimental validation.

In the present study, these findings were integrated with previous work, supplemented by additional confirmatory experiments, and compiled for publication in an international journal. This report represents one such effort. It is anticipated that the experimental findings presented here will contribute to future research aimed at the development of therapeutic and preventive strategies for glomerulonephritis.
